# Two-year results of Lenslet-ARray-Integrated spectacle lenses for myopia control in children

**DOI:** 10.1186/s40662-025-00462-0

**Published:** 2025-11-01

**Authors:** Binbin Su, Pauline Cho, Stephen J. Vincent, Jingwei Zheng, Jiaojie Chen, Huiling Lin, Feifu Wang, Zihao Sheng, Xueqian Wang, Fan Lu, Jun Jiang

**Affiliations:** 1 National Clinical Research Center for Ocular Diseases, Eye Hospital, Wenzhou Medical University, Wenzhou, 325027 China; 2 Department of Ophthalmology and Vision Science, Eye and ENT Hospital, Fudan University, 83 Fenyang Road, Shanghai, 200031 China; 3 Department of Ophthalmology, West China Hospital, Sichuan University, Chengdu, China; 4 Optometry and Vision Science, Centre for Vision and Eye Research, Queensland University of Technology, Brisbane, QLD Australia; 5 Eye Hospital and School of Ophthalmology and Optometry, Wenzhou Medical University, 270 Xueyuan Road, Wenzhou, Zhejiang 325003 People’s Republic of China

**Keywords:** Myopia control, Axial elongation, Myopic defocus, Hyperopic defocus, Power lenslet

## Abstract

**Purpose:**

To investigate the 2-year myopia control efficacy of Lenslet-ARray-Integrated (LARI) lenses with positive (PLARI) and negative (NLARI) power lenslets and the effect of switching lens designs.

**Methods:**

A total of 218 children, who were randomly assigned to wear PLARI, NLARI, or single-vision (SV) lenses in Phase 1 continued in this randomized, double-masked extended trial for an additional year (Phase 2). Participants were randomly assigned to one of six groups: SV to PLARI, SV to NLARI, PLARI to PLARI (P-PLARI), PLARI to NLARI (P-NLARI), NLARI to PLARI (N-PLARI), and NLARI to NLARI (N-NLARI). In year 2, the change in spherical equivalent refraction (SER) and axial elongation (AE) from the SV group were extrapolated based on published data [the extrapolated single vision (ESV) group]. Linear models were used to determine differences in SER changes and AE among groups in 2 years and in Phase 2 only.

**Results:**

After 2 years, the SER changes (− 0.87 ± 0.68 D, − 0.64 ± 0.86 D, − 0.68 ± 0.54 D, and − 0.75 ± 0.62 D, respectively) and AE (0.44 ± 0.33 mm, 0.33 ± 0.32 mm, 0.36 ± 0.23 mm, and 0.39 ± 0.25 mm, respectively) of P-PLARI, P-NLARI, N-PLARI, and N-NLARI were significantly smaller than those in the ESV group (SER: − 1.24 ± 0.77 D, all *P* < 0.05; AE: 0.63 ± 0.33 mm, all *P* < 0.001). In Phase 2, there was no significant difference in SER changes among the four LARI groups and ESV group (*P* = 0.58). In Phase 2, AE of the P-NLARI and N-PLARI groups was significantly smaller than the ESV group (*P* < 0.001 and *P* = 0.001), and AE of the P-PLARI and N-NLARI groups were slightly smaller than that of ESV group (*P* = 0.054 and *P* = 0.10), but there were no significant differences in AE among the four LARI groups (all *P* > 0.05).

**Conclusions:**

Wearing LARI lenses for 2 years effectively slowed myopia progression and AE. Switching to another LARI design after 1 year improved myopia control efficacy, in terms of AE, during the second year, but not SER progression.

**Trial registration:**

Chinese Clinical Trial Registry, ChiCTR2200057210. Registered 03 March 2022, https://www.chictr.org.cn/bin/project/edit?pid=152900.

**Supplementary Information:**

The online version contains supplementary material available at 10.1186/s40662-025-00462-0.

## Background

With its increasing prevalence and younger age of onset, myopia has become a global public health concern [1]. It has been predicted that by 2050, the global prevalence of myopia among children and adolescents will increase to 40%, and approximately 740 million children and adolescents will be affected [2]. Therefore, slowing childhood myopia progression is gaining increasing attention [3].

Currently, the main optical interventions to control myopia include orthokeratology [4, 5], multifocal contact lenses [5, 6], and myopia control spectacle lenses [7–9], which are all effective in controlling myopia progression [10, 11]. Nevertheless, in clinical practice, there are still some issues to be addressed with these interventions, such as improving long-term efficacy. Previous studies have shown that the myopia control efficacy of optical interventions is greater in the first year and decreases with time [12–14]. The reason why the efficacy of myopia control treatments decrease over time is still unclear. A possible explanation is that the human retina has neural adaptability [15], and the prolonged use of the same optical intervention may trigger an adaptive response, leading to a decrease in the retina’s sensitivity to the optical signals that slow myopia progression. Therefore, further research is needed to understand whether changing the optical signals during long-term myopia control can slow this temporal reduction in efficacy.

The Lenslet-ARray-Integrated (LARI) spectacle lenses are novel myopia control lenses that are available in two designs, with either + 3.00 D (PLARI) or − 3.00 D (NLARI) lenslets affixed to the peripheral region of the lenses, as described in detail previously [16]. The first-year LARI study (Phase 1) demonstrated that both LARI designs had significant myopia control effects, with axial elongation (AE) reductions of 0.15 mm and 0.17 mm [17]. This study reports Phase 2 of the LARI study, with the following aims: (1) to determine the myopia control effect of wearing LARI lenses for a second year by comparing PLARI and NLARI groups with an extrapolated single-vision (ESV) spectacle lens group; (2) to compare the myopia control effect between those who wore the same design LARI lenses in Phases 1 and 2 with those who switched to the other design in Phase 2.

## Methods

### Study design

The Phase 1 study design was described previously [17], where participants (6 to 12 years) with spherical equivalent refraction (SER) of − 4.00 to − 1.00 D, astigmatism ≤ 1.50 D, and anisometropia ≤ 1.00 D, were randomly assigned to single-vision (SV), PLARI, and NLARI groups and followed for 12 months [17].

**Fig. 1:**
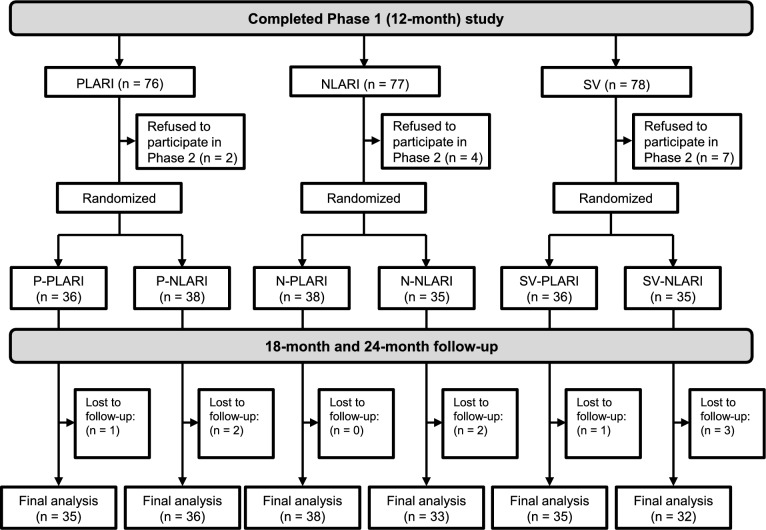
Phase 2 study design and participants at each visit. PLARI and NLARI, Lenslet-ARray-Integrated spectacle lens with lenslets of + 3.00 D and − 3.00 D addition powers, respectively; SV, single-vision spectacle lens; SV-PLARI and SV-NLARI, switched over to PLARI and NLARI, respectively in Phase 2; P-PLARI and N-NLARI, continued PLARI and NLARI, respectively in Phase 2; P-NLARI and N-PLARI, switched over to NLARI and PLARI, respectively in Phase 2

Participants who completed Phase 1 were invited to continue in Phase 2 (randomized and double masked) of the LARI study at the Wenzhou Medical University Affiliated Eye Hospital from July 2023 to August 2024. After completing the 12-month follow-up, the 240 participants who were initially randomly assigned to Phase 1 were unmasked, and then a spreadsheet generator (Excel; Microsoft) was used to randomly reassign the participants in the SV, PLARI, and NLARI groups in a 1:1 ratio, respectively. Random sequences were generated by a statistician and presented in a central random system for researchers to use. Since the Phase 1 data confirmed that both LARI lens designs provided better myopia control than SV lenses [17], all participants in the SV group were randomized to switch to either PLARI lenses (SV-PLARI group) or NLARI lenses (SV-NLARI group) in Phase 2 in a 1:1 ratio, for ethical reasons. The participants who wore PLARI and NLARI lenses in Phase 1 were randomly assigned, at a 1:1 ratio within the group, to either continue wearing LARI lenses of the same design in Phase 2, that is, PLARI to PLARI, NLARI to NLARI (P-PLARI and N-NLARI groups, respectively) or switch to the other design (P-NLARI and N-PLARI groups, respectively) (see Fig. 1). After randomization in Phase 2, both the researchers and participants were masked to the group allocation until the end of the trial.

The study was registered with the Chinese Clinical Trial Registry (ChiCTR2200057210) and approved by the ethics committee of the Eye Hospital of Wenzhou Medical University (2021-241-K-211-03). All procedures were conducted in accordance with the tenets of the Declaration of Helsinki and written informed consent was obtained from participants and their parents.

### Interventions

The design of the LARI lens has been described in detail in previous studies [16, 17]. Briefly, the LARI lens consists of an 8 mm diameter central zone incorporating the distance refractive error correction and a control zone composed of a non-coaxial lenslet array. The PLARI design contains a + 3.00 D lenslet array, and the NLARI design contains a − 3.00 D lenslet array. LARI lenses are resin lenses produced by Shanghai Gino Optical Spectacle Co. Ltd. and were offered to the participants at no charge.

### ESV group

The meta-analysis results of Smotherman et al. and Brennan et al. estimated that SER progression and AE decreased by 9.7% [18] and 15% [19] per year respectively, for this age group. This approach has recently been leveraged in two clinical studies [20, 21]. Using this method, the SER progression and AE of the ESV group in Phase 2 were extrapolated from the data of the SV group in Phase 1 of the LARI study.

### Study procedures and data collection

The ophthalmic examinations and other data collection in Phase 2 were the same as in Phase 1, as described previously [17]. In brief, ocular parameters, compliance, and adverse events were assessed every 6 months. Axial length was measured using the IOLMaster 700 (Carl Zeiss Meditec AG, Jena, Germany) before cycloplegia, and the average of five measurements was recorded. Cycloplegic objective refraction was measured with a closed-field autorefractor (Topcon KR-8800; Topcon Corporation, Japan), and the average of three SER measurements (with differences within 0.25 D) was recorded for analysis. Cycloplegia was induced using two drops of 1% cyclopentolate (Alcon-Couvreur SA, Belgium) with a 5 min interval between each drop, followed by objective refraction at least 30 min after the second drop of cyclopentolate. Intraocular pressure was measured using noncontact tonometry (Canon TX-20; Canon, Inc., Tokyo, Japan) and corneal curvature was measured with the Medmont E300 corneal topographer (Medmont International Pty Ltd., Victoria, Australia). Like Phase 1, Phase 2 also required participants (or parents) to report lens-wearing time (hours per day, days per week) at each follow-up. The average daily wearing hours in Phase 2 was calculated using the following formula: (total wearing hours per week reported at 18 months + total wearing hours per week reported at 24 months) ÷ 2 (periods) ÷ 7 (days). The age of myopia onset was obtained from the outpatient system of the hospital or from the children and their guardians.

As in Phase 1, spectacle prescriptions were updated if the SER increased by 0.50 D or more at any data collection visit.

### Statistical analyses

Statistical analyses were performed using SPSS statistics software version 26 (IBM Corp., Armonk, NY, USA) and GraphPad Prism 8 (GraphPad Software Inc, Boston, MA, USA). Only data from the right eye of participants who completed the 2-year follow-up were included in the analysis. Changes in ocular parameters were determined for analyses. The Wilcoxon rank sum test was used for ordered categorical variables, and the Chi-squared test or Fisher’s exact probability test was used for unordered categorical variables. Unpaired t-tests were used to test for differences in demographics and characteristics of participants at the beginning of the Phase 2 between SV-PLARI and SV-NLARI, P-PLARI and P-NLARI, N-PLARI and N-NLARI groups. Linear mixed-effects models were used to determine differences in SER changes and AE among groups after adjusting for initial age, sex, SER or axial length, age of myopia onset, and number of parents with myopia. A two-tailed *P* value < 0.05 was considered statistically significant.

## Results

In total, 218 participants (90.8%) from Phase 1 continued to Phase 2 (Fig. 1), of which 209 (87.1%) completed the study. The demographics and characteristics of the groups at the beginning of Phase 2 were similar, except that the SER in the SV-PLARI group was more myopic than the SV-NLARI group; axial length in the P-PLARI group was shorter than that in the P-NLARI group, and age of participants was older as well as SER was more myopic in the N-NLARI group than in the N-PLARI group (Table 1). The ESV group included 67 participants who had worn SV lenses for 1 year in Phase 1 of the LARI study (Table 1). Baseline characteristics of participants who completed the 2-year study were similar to those who did not (n = 31, Table S1).

**Table 1:** Demographics and characteristics of participants at the beginning of Phase 2 (who completed both phases of the study)

Demographics/Parameter	ESV	SV (n = 67)			PLARI (n = 71)			NLARI (n = 71)		
		SV-PLARI	SV-NLARI	*P* value	P-PLARI	P-NLARI	*P* value	N-PLARI	N-NLARI	*P* value
N	67	35	32		35	36		38	33	
Age (years)	10.9 ± 1.6	11.1 ± 1.6	10.6 ± 1.7	0.25	10.4 ± 1.5	10.6 ± 1.6	0.63	10.3 ± 1.6	11.0 ± 1.3	**0.03**
Sex* (Male, %)	38 (56.7)	22 (62.9)	16 (50.0)	0.29^‡^	14 (40.0)	21 (58.3)	0.12^‡^	18 (47.4)	18 (54.5)	0.55 ^‡^
SER (D)	− 2.86 ± 0.70	− 3.04 ± 0.65	− 2.66 ± 0.71	**0.03**	− 2.68 ± 0.73	− 2.51 ± 0.87	0.39	− 2.28 ± 0.75	− 2.72 ± 0.85	**0.02**
Phase 1 SER changes (D)	− 0.65 ± 0.40	− 0.61 ± 0.37	− 0.69 ± 0.45	0.46	− 0.34 ± 0.39	− 0.24 ± 0.53	0.35	− 0.20 ± 0.37	− 0.22 ± 0.36	0.82
Axial length (mm)	24.92 ± 0.74	25.01 ± 0.61	24.82 ± 0.87	0.31	24.49 ± 0.84	24.82 ± 0.49	**0.049**	24.52 ± 0.96	24.70 ± 0.98	0.43
Phase 1 axial elongation (mm)	0.34 ± 0.18	0.33 ± 0.15	0.35 ± 0.20	0.62	0.21 ± 0.19	0.16 ± 0.21	0.26	0.16 ± 0.15	0.18 ± 0.15	0.43
Age at myopia onset (years) (self-reported)	8.9 ± 1.6	9.0 ± 1.7	8.8 ± 1.5	0.63	8.4 ± 1.7	8.5 ± 1.7	0.91	8.5 ± 1.7	8.9 ± 1.2	0.17
Best-corrected visual acuity (logMAR)	− 0.06 ± 0.06	− 0.05 ± 0.06	− 0.06 ± 0.06	0.42	− 0.05 ± 0.05	− 0.06 ± 0.05	0.42	− 0.06 ± 0.05	− 0.05 ± 0.05	0.45
Intraocular pressure (mmHg)	15.93 ± 1.95	15.93 ± 1.73	15.93 ± 2.20	0.99	16.08 ± 2.41	15.94 ± 1.89	0.79	16.65 ± 1.89	16.28 ± 1.80	0.40
Myopic parents* (n, %)				0.88 ^†^			0.56^†^			0.28 ^†^
0	23 (34.3)	13 (37.1)	10 (31.3)		6 (17.1)	3 (8.3)		9 (23.7)	4 (12.1)	
1	20 (29.9)	9 (25.7)	11 (34.4)		17 (48.6)	20 (55.6)		15 (39.5)	14 (42.4)	
2	24 (35.8)	13 (37.1)	11 (34.4)		12 (34.3)	13 (36.1)		14 (36.8)	15 (45.5)	

*N* = number of participants; *SV* = single-vision spectacle lens group; *ESV* = the extrapolated single-vision spectacle lenses group proposed by Brennan et al. [19] and Smotherman et al. [18] in Phase 2; *PLARI and NLARI *= Lenslet-ARray-Integrated spectacle lens with lenslets of + 3.00 D and − 3.00 D addition powers, respectively;* SV-PLARI* *and SV-NLARI* = switched over to PLARI and SLARI, respectively in Phase 2; *P-PLARI and N-NLARI* = continued PLARI and NLARI, respectively in Phase 2; *P-NLARI and N-PLARI* = switched over to NLARI and PLARI, respectively in Phase 2; *SER* = spherical equivalent refraction; *logMAR* = logarithm of the minimum angle of resolution

Data presented as mean ± standard deviation; * number (%). Boldface values indicate statistical significance

*P* are probability values of unpaired t-test, except those marked ^†^(Wilcoxon rank sum test), and ^‡^(Chi-squared test)

The age at myopia onset was obtained from the outpatient system of the hospital or from the children and their guardians

### Refractive changes and AE in the LARI groups in Phase 2

In Phase 2, there were no statistically significant differences in adjusted SER changes among the four LARI and ESV groups (*P* = 0.58; Table 2). AE in the P-NLARI and N-PLARI groups was significantly less than that in ESV group, with adjusted differences of 0.11 mm (95% CI: 0.04 to 0.19 mm, *P* < 0.001) and 0.10 mm (95% CI: 0.03 to 0.18 mm, *P* = 0.001; Table 2). However, AE in the P-PLARI and N-NLARI groups, did not differ statistically from the ESV group (Table 2). Additionally, no statistically significant differences in adjusted AE were observed among the four LARI groups (all *P* > 0.05).

**Table 2:** Changes in spherical equivalent refraction and axial elongation in all participants who completed Phase 2

Parameter	SV/ESV (n = 67)	P-PLARI (n = 35)	P-NLARI (n = 36)	N-PLARI (n = 38)	N-NLARI (n = 33)	*P* value	*P* value (ESV vs. P-PLARI, ESV vs. P-NLARI, ESV vs. N-PLARI, ESV vs. N-NLARI)
SER changes (D)							
Phases 1 & 2	− 1.24 ± 0.77	− 0.87 ± 0.68	− 0.64 ± 0.86	− 0.68 ± 0.54	− 0.75 ± 0.62	< 0.001*	**0.049**, < **0.001**, < **0.001**,** 0.04**
Phase 1	− 0.65 ± 0.4	− 0.34 ± 0.39	− 0.24 ± 0.53	− 0.20 ± 0.37	− 0.22 ± 0.36	< 0.001*	**0.002**, < **0.001**, < **0.001**, < **0.001**
Phase 2	− 0.59 ± 0.37	− 0.53 ± 0.45	− 0.41 ± 0.43	− 0.48 ± 0.33	− 0.53 ± 0.39	0.58*	1.00, 0.80, 0.99, 1.00
Axial elongation (mm)							
Phases 1 & 2	0.63 ± 0.33	0.44 ± 0.33	0.33 ± 0.32	0.36 ± 0.23	0.39 ± 0.25	** < 0.001**^†^	**0.001**, < **0.001**, < **0.001**,** 0.001**
Phase 1	0.34 ± 0.18	0.21 ± 0.19	0.16 ± 0.21	0.16 ± 0.15	0.18 ± 0.15	** < 0.001**^†^	** < 0.001**, < **0.001**, < **0.001**,** < 0.001**
Phase 2	0.29 ± 0.15	0.22 ± 0.16	0.18 ± 0.13	0.21 ± 0.13	0.20 ± 0.14	** < 0.001**^†^	0.054, < **0.001**, **0.001**, 0.10

*SV* = single-vision spectacle lens group; *ESV* = the extrapolated single-vision spectacle lenses group proposed by Brennan et al. [19] and Smotherman et al. [18] in Phase 2; *PLARI and NLARI* = Lenslet-ARray-Integrated spectacle lens with lenslets of + 3.00 D and − 3.00 D addition powers, respectively; *P-PLARI and N-NLARI* = continued PLARI and NLARI, respectively in Phase 2; *P-NLARI and N-PLARI* = switched over to NLARI and PLARI, respectively in Phase 2; *SER* = spherical equivalent refraction

Data are presented as mean ± standard deviation, unless otherwise indicated. Boldface values indicate statistical significance

^*^Probability values of linear mixed-effect models with age, sex, age at myopia onset, the number of parents with myopia, and initial SER adjustment for SER comparisons

^†^Probability values of linear mixed-effect models with age, sex, age at myopia onset, the number of parents with myopia, and initial axial length adjustment for axial elongation comparisons

### Refractive changes and AE over two years in the LARI groups

**Fig. 2:**
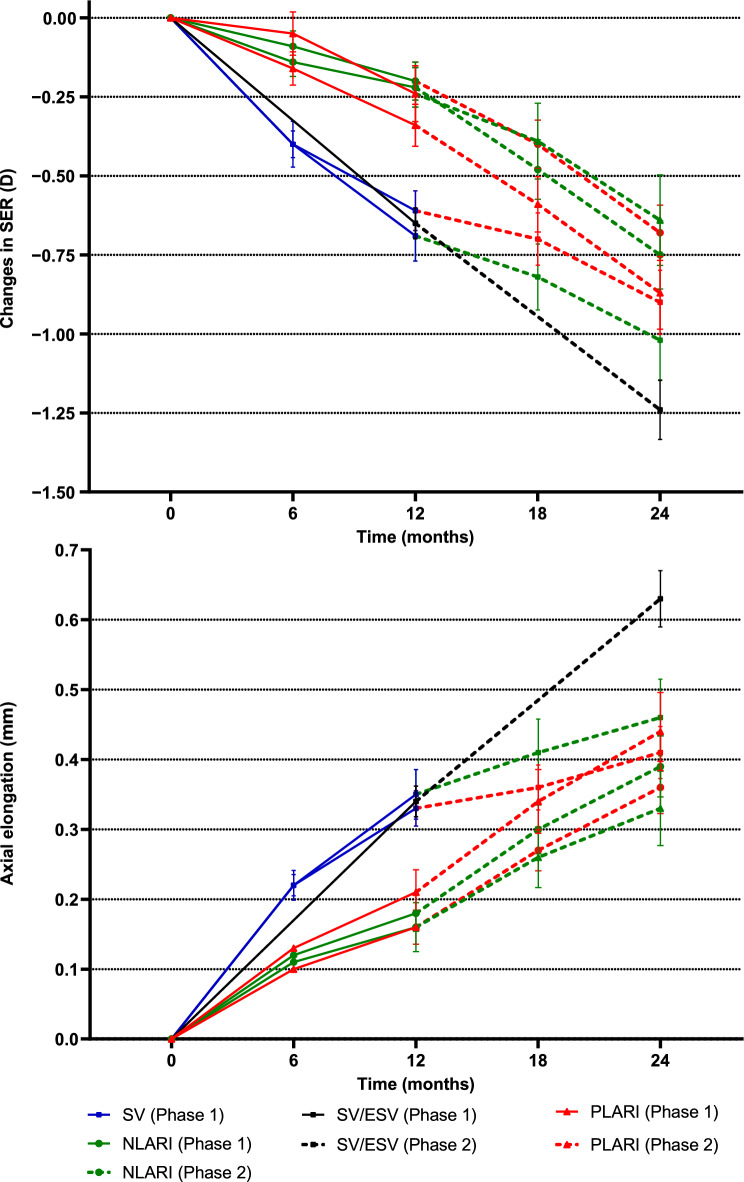
Mean changes in spherical equivalent refraction (SER) (**a**) and axial elongation (**b**) of participants wearing Lenslet-ARray-Integrated (LARI) lenses in Phase 2 (solid line—changes observed in Phase 1). SV, single-vision; PLARI and NLARI, Lenslet-ARray-Integrated spectacle lens with lenslets of + 3.00 D and − 3.00 D addition powers, respectively; ESV, extrapolated single-vision. Error bars are standard errors

The 2-year SER changes and AE in the P-PLARI, P-NLARI, N-PLARI, and N-NLARI groups were significantly less than those in the ESV group, with adjusted mean differences for SER changes of 0.41 D (95% CI: 0.00 to 0.82 D, *P* = 0.049), 0.61 D (95% CI: 0.20 to 1.02 D, *P* < 0.001), 0.64 D (95% CI: 0.25 to 1.04 D, *P* < 0.001), and 0.43 D (95% CI: 0.01 to 0.84 D, *P* = 0.04), and for AE of 0.22 mm (95% CI: 0.07 to 0.38 mm, *P* = 0.001), 0.32 mm (95% CI: 0.16 to 0.48 mm, *P* < 0.001), 0.32 mm (95% CI: 0.16 to 0.47 mm, *P* < 0.001), and 0.23 mm (95% CI: 0.07 to 0.38 mm, *P* = 0.001), respectively (Fig. 2a and 2b**; **Table 2). The SER changes and AE in the P-PLARI and N-NLARI groups were slightly greater than those in the P-NLARI and N-PLARI groups, but the adjusted differences among the four groups were not statistically significant (all *P* > 0.05; Table 2).

### Association factors of annual refractive changes and AE in the LARI groups

Among the participants who wore LARI lenses for 2 years, the annual SER changes were negatively correlated with the year of treatment and positively correlated with age, but not with the treatment groups (Table 3). The annual AE was positively correlated with the year of treatment and negatively correlated with age, but not with the treatment groups (Table 3).

**Table 3:** Mixed-effects models of annual spherical equivalent refraction/axial length changes in the 2-year LARI groups

	Annual SER changes			Annual axial elongation		
Parameter	Beta (β)	95% CI	*P* value	Beta (β)	95% CI	*P* value
Treatment year (first year as reference)	− 0.32	− 0.42 to − 0.22	** < 0.001**	0.07	0.03 to 0.10	** < 0.001**
Age (years)	0.08	0.01 to 0.15	**0.03**	− 0.04	− 0.06 to − 0.01	**0.003**
Sex (male as reference)	0.01	− 0.10 to 0.13	0.80	− 0.03	− 0.08 to 0.02	0.19
Age at myopia onset (years)	− 0.02	− 0.09 to 0.05	0.54	0.003	− 0.02 to 0.03	0.81
Myopic parents						
0	Ref			Ref		
1	0.06	− 0.11 to 0.23	0.52	− 0.005	− 0.07 to 0.06	0.88
2	0.004	− 0.17 to 0.18	0.96	− 0.002	− 0.07 to 0.06	0.96
SER at the beginning of one year (D)	− 0.03	− 0.11 to 0.04	0.42	-	-	-
Axial length at the beginning of one year (mm)	–	–	–	− 0.01	− 0.04 to 0.02	0.42
Treatment groups						
P-PLARI	Ref			Ref		
P-NLARI	0.10	− 0.06 to 0.26	0.22	− 0.04	− 0.10 to 0.02	0.17
N-PLARI	0.12	− 0.04 to 0.28	0.15	− 0.03	− 0.09 to 0.03	0.29
N-NLARI	0.02	− 0.15 to 0.18	0.85	0.0002	− 0.06 to 0.06	1.00

*LARI* = Lenslet-ARray-Integrated; *PLARI and NLARI* = LARI spectacle lens with lenslets of + 3.00 D and − 3.00 D addition powers, respectively; *P-PLARI and N-NLARI *= continued PLARI and NLARI, respectively in Phase 2; *P-NLARI and N-PLARI *= switched over to NLARI and PLARI, respectively in Phase 2; *SER* = spherical equivalent refraction

Boldface values indicate statistical significance

### Refractive changes and AE in the SV-PLARI and SV-NLARI groups

In Phase 2, the SER changes and AE in the SV-PLARI and SV-NLARI groups were significantly less than those in the ESV group, with adjusted mean differences for SER changes of 0.32 D (95% CI: 0.15 to 0.49 D, *P* < 0.001) and 0.23 D (95% CI: 0.06 to 0.41 D, *P* = 0.004), and for AE of 0.20 mm (95% CI: 0.14 to 0.26 mm, *P* < 0.001) and 0.19 mm (95% CI: 0.13 to 0.25 mm, *P* < 0.001), respectively (Table S2). The SER changes and AE between SV-PLARI and SV-NLARI groups were similar, and there were no statistically significant differences (*P* = 0.66 and *P* = 0.97, respectively).

### Distribution of participants with myopia progression

**Fig. 3:**
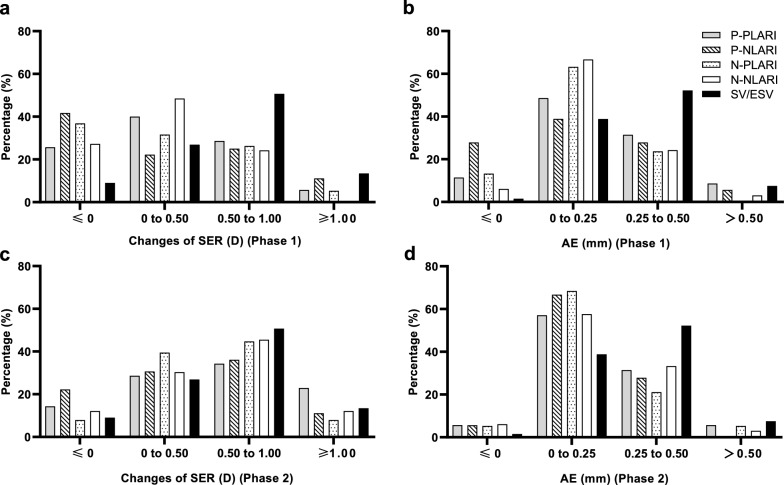
Distributions of changes in spherical equivalent refraction (SER) (**a**, **c**) and axial elongation (**b**, **d**) in Phases 1 and 2. PLARI and NLARI, Lenslet-ARray-Integrated spectacle lens with lenslets of + 3.00 D and − 3.00 D addition powers, respectively; P-PLARI and N-NLARI, continued PLARI and NLARI, respectively in Phase 2; P-NLARI and N-PLARI, switched over to NLARI and PLARI, respectively in Phase 2; ESV, extrapolated single-vision

In Phase 2, the percentages of participants with SER changes ≤ 0.50 D in the four LARI groups were lower than those observed in Phase 1 (Figs. 3a and 3c, Table S3). The percentages of participants in these four LARI groups with AE ≤ 0.25 mm in Phase 2 were similar to those in Phase 1 (Figs. 3b and 3d, Table S3). A total of 29 participants (20.4%) had AE > 0.25 mm in both Phases. Only four participants (2.8%) had AE ≤ 0 mm in both phases.

### Compliance and adverse events

The average daily wearing hours of each group in Phase 2 were significantly longer than those in Phase 1 (all *P* ≤ 0.01; Table S4). The average daily wearing hours of the N-PLARI group (13.7 ± 1.0 h) was significantly shorter than SV-PLARI (14.3 ± 0.7 h) and P-NLARI groups (14.3 ± 0.9 h) (*P* = 0.04 and *P* = 0.03, respectively). No significant differences were found among the other groups (all *P* > 0.30).

No statistically significant differences were found among the six groups in terms of the changes in best-corrected visual acuity, intraocular pressure, corneal curvature, or corneal astigmatism in Phases 1 and 2 (Table S5). During the 2-year follow-up, no adverse events related to the intervention were reported.

## Discussion

The current study demonstrated that, compared to the ESV group, wearing LARI lenses for 2 years significantly slowed myopia progression and AE. However, the myopia control effect decreased in the second year, especially for SER. Compared with continually using the same LARI lens design, switching to another design after one year did result in less AE relative to the ESV group during the second year, but no effect was observed for SER progression.

Previous studies have reported that wearing spectacle lenses with peripheral positive power lenslets for 2 years had a significant myopia control effect (Table S6) [7, 8, 22]. Similarly, the results of the current study showed that compared with the ESV group, myopia progression was significantly reduced in the P-PLARI group by 0.37 D and AE by 0.19 mm. The myopia control efficacy of PLARI over 2 years was less than observed for defocus incorporated multiple segments (DIMS) [8] and highly aspherical lenslets (HAL) [7] in clinical trials, but comparable to the efficacy of DIMS under clinical settings [22]. Differences in lens design (such as lenslet power and distribution), research methodologies, and the age of participants may account for the differences across studies. In addition, the current study also confirmed that, similar to the PLARI lens, wearing the NLARI lens for 2 years still had a significant myopia control effect. In tandem with the results from Phase 1 [17], significant myopia control effects were observed with both types of LARI lenses (i.e., with either positive or negative power lenslets), suggesting the workings of other mechanisms for myopia control beyond the traditional defocus theory (i.e., that imposed hyperopic defocus results in AE).

Data from clinical trials have shown that the myopia control efficacy of optical interventions decreases with time [13]. In a meta-analysis of multifocal spectacles for myopia control, Kaphle et al. reported that the greatest myopia control effect was in the first 6– 12 months of treatment [12]. The current study showed that in the participants who wore LARI lenses for 2 years, SER progression in Phase 2 resembled that in the ESV group in Phase 2 and was faster than those in the LARI groups in Phase 1. This indicates that the myopia control effect for SER progression was reduced in the second year. However, for AE, the results of all four LARI groups in Phase 2 were still 0.07 – 0.11 mm slower than those in the ESV group. The magnitudes of SER changes in Phase 2 across all LARI groups were approximately double those observed in Phase 1. However, AE in Phase 2 saw only about a 15% increase. This discrepancy may be related to changes in physiological eye growth with ageing [13]. Liu et al. reported that the SER progression/AE ratio varies with age and refractive status, with greater changes observed in SER relative to AE in older children [23].

Clinically, some children still progress rapidly after treatment with an optical intervention, and practitioners may try to improve the effect of myopia control by switching to a different intervention. Li et al. reported that in participants who switched from slightly aspherical lenslets (SAL) to HAL, SER changes after switching treatment were not significantly different from those in the previous year, whereas AE decreased compared with the previous year [24]. However, to date, there is insufficient evidence showing that switching to different treatment modalities is an effective strategy for myopia control [25, 26]. In the current study, there were no significant differences in SER progression and AE in the second year and over the entire 2 years between participants who wore LARI lenses of the same design for two consecutive years, and those who wore a different LARI design each year. However, the magnitudes of AE in Phase 2 for the P-NLARI and N-PLARI groups were significantly less than the ESV group, while those for the P-PLARI and N-NLARI groups were comparable to the ESV group. This suggests that switching between LARI lens designs after 1 year slightly improved myopia control in terms of AE during the second year but did not significantly affect SER progression. Although there are significant differences in the optical design of the two LARI lenses, with + 3.00 D and − 3.00 D additional power lenslet arrays, respectively, the point spread function, image simulation, and modulation transfer function performance of the two LARI lenses are similar [16]. The similar modulation of retinal imaging quality by PLARI and NLARI may explain why their myopia control effects are comparable [17]. Future studies should explore whether switching to wearing spectacle lenses which modulate retinal image quality to varying degrees can improve long-term myopia control efficacy.

Lam et al. found that the SER changes in children wearing DIMS lenses were related to age and the SER changes in older children were smaller [8]. In a clinical trial involving SAL and HAL lenses, Bao et al. reported that AE in children in the SAL group was negatively correlated with age, while AE in children in the HAL group was not [27]. The current study found that in the 2-year LARI participants, the annual SER changes and AE were significantly correlated with age, but not with the treatment groups. Age is an important factor affecting myopia control, and while switching to wearing another LARI design in the second year slightly improved AE relative to the ESV group compared to continuing with the same design, there was no effect on SER progression. Therefore, for children who exhibit a poor response to myopia control spectacle lenses in clinical practice, it may be necessary to switch to more effective myopia control methods, such as combining low concentration atropine [28] or switching to orthokeratology [29–31].

A limitation of this study was that all participants in the control group from Phase 1 were switched to PLARI or NLARI lenses during Phase 2. This was due to ethical considerations, as both PLARI and NLARI lenses had significant myopia control effects compared to SV lenses. Therefore, myopia control effects of LARI lenses relative to SV lenses over 2 years could not be compared. However, to minimize this limitation, as suggested by Smotherman et al. [18] and Brennan et al. [19], data from the SV group in Phase 1 was extrapolated as the control group (i.e., the ESV group) in Phase 2 for comparison. In addition, in-group secondary randomization was utilized in the second year, resulting in a smaller sample size for each group. Future randomized controlled clinical trials with larger sample sizes may be needed to further validate the myopia control efficacy in each sub-group.

## Conclusions

Compared with the ESV group, wearing LARI spectacle lenses with positive and negative power lenslets for 2 years effectively slowed myopia progression in Chinese myopic children. However, compared to the first year of wear, the myopia control effect on SER significantly decreased in the second year. Switching to another LARl design after one year of treatment resulted in smaller AE, but not SER, when compared to the ESV group during the second year.

## Supplementary Information

Additional file 1.

## Data Availability

The data that support the findings of this study are available from the corresponding author upon reasonable request.

## Abbreviations

SER: Spherical equivalent refraction
AE: Axial elongation
DIMS: Defocus incorporated multiple segments
HAL: Highly aspherical lenslets
SAL: Slightly aspherical lenslets
SV: Single vision
ESV: Extrapolated single-vision
LARI: Lenslet-ARray-Integrated
PLARI: Lenslet-ARray-Integrated spectacle lens with lenslets of + 3.00 D addition power
NLARI: Lenslet-ARray-Integrated spectacle lens with lenslets of − 3.00 D addition power
SV-PLARI: SV switched over to PLARI in Phase 2
SV-NLARI: SV switched over to NLARI in Phase 2
P-PLARI: PLARI continued PLARI in Phase 2
N-NLARI: NLARI continued NLARI in Phase 2
P-NLARI: PLARI switched over to NLARI in Phase 2
N-PLARI: NLARI switched over to PLARI in Phase 2
