# Supplementary material for: Two-year results of Lenslet-ARray-Integrated spectacle lenses for myopia control in children
Source: Eye Vis (Lond). 2025 Nov 1;12:45. doi: 10.1186/s40662-025-00462-0 (PMC12579422; doi:10.1186/s40662-025-00462-0)
Supplement: Supplementary file 1 — Additional file 1. [file 40662_2025_462_MOESM1_ESM.docx]

| **Supplementary Material**  **Table S1**. Demographics and ocular parameters of included and excluded participants. | | | | | | | | |
| --- | --- | --- | --- | --- | --- | --- | --- | --- |
| **Demographics/Parameter** | | | **Included**  **(n = 209)** | | **Excluded**  **(n = 31)** | ***P*** | | |
| Age (years) | | | 9.6 ± 1.6 | | 9.7 ± 1.8 | 0.92 | | |
| Sex (Male, %) | | | 109 (52.2) | | 15 (48.4) | 0.70 ^†^ | | |
| SER (D) | | | −2.26 ± 0.74 | | −2.28 ± 0.88 | 0.93 | | |
| Axial length (mm) | | | 24.49 ± 0.80 | | 24.39 ± 0.89 | 0.50 | | |
| Best-corrected visual acuity (logMAR) | | | 5.03 ± 0.05 | | 5.03 ± 0.04 | 0.64 | | |
| Intraocular pressure (mmHg) | | | 14.7 ± 2.2 | | 14.6 ± 2.3 | 0.83 | | |
| Flat keratometry (D) | | | 42.85 ± 1.48 | | 43.16 ± 1.37 | 0.28 | | |
| Steep keratometry (D) | | | 43.92 ± 1.55 | | 44.34 ± 1.49 | 0.15 | | |
| Corneal astigmatism (D) | | | 1.07 ± 0.42 | | 1.19 ± 0.41 | 0.14 | | |
| Age at myopia onset (years) (self-reported) | | | 8.7 ± 1.6 | | 8.9 ± 1.8 | 0.57 | | |
| Myopic parents (n, %) * | | |  | |  | 0.72 ^‡^ | | |
| 0 | | | 45 (21.5) | | 4 (12.9) |  | | |
| 1 | | | 86 (41.1) | | 13 (41.9) |  | | |
| 2 | | | 78 (37.3) | | 10 (32.3) |  | | |
| SER = spherical equivalent refraction; logMAR = logarithm of the minimum angle of resolution.  Data presented as mean ± standard deviation.  * Four excluded participants did not provide the relevant information.  *P* are probability values of unpaired t-tests, except those marked ^†^ (Chi-squared test) and ^‡^ (Wilcoxon rank-sum test). | | | | | | | | |
| **Table S2**. Changes in spherical equivalent refraction and axial elongation of participants in SV group who completed Phase 2. | | | | | | | | |
| **Parameter** | **SV/ESV**  **(n = 67)** | | **SV-PLARI**  **(n = 35)** | **SV-NLARI**  **(n = 32)** | | ***P* value** | ***P* value (ESV vs SV-PLARI, ESV vs SV-NLARI, SV-PLARI vs SV-NLARI)** | |
| SER changes (D) |  | |  |  | |  |  | |
| Phases 1 & 2 | −1.24 ± 0.77 | | −0.90 ± 0.60 | −1.02 ± 0.72 | | 0.06 * | 0.14, 0.16, 1.00 | |
| Phase 1 | −0.65 ± 0.4 | | −0.61 ± 0.37 | −0.69 ± 0.45 | | 0.99 * | 1.00, 1.00, 1.00 | |
| Phase 2 | −0.59 ± 0.37 | | −0.28 ± 0.37 | −0.34 ± 0.39 | | **< 0.001 *** | **< 0.001**, **0.004**, 0.97 | |
|  |  | |  |  | |  |  | |
| Axial elongation (mm) |  | |  |  | |  |  | |
| Phases 1 & 2 | 0.63 ± 0.33 | | 0.41 ± 0.22 | 0.46 ± 0.31 | | **< 0.001** ^†^ | **< 0.001**, **0.001**, 0.99 | |
| Phase 1 | 0.34 ± 0.18 | | 0.33 ± 0.15 | 0.35 ± 0.20 | | 0.99 ^†^ | 1.00, 1.00, 1.00 | |
| Phase 2 | 0.29 ± 0.15 | | 0.08 ± 0.12 | 0.11 ± 0.15 | | **< 0.001** ^†^ | **< 0.001**, **0.004**, 0.66 | |
|  |  | |  |  | |  |  | |
| SV = single-vision spectacle lens group; ESV = the extrapolated single-vision spectacle lenses group proposed by Brennan et al. [19] and Smotherman et al [18] in Phase 2; SV-PLARI and SV-NLARI = switched over to Lenslet-ARray-Integrated spectacle lens with lenslets of +3.00 D and −3.00 D addition powers, respectively in Phase 2; SER = spherical equivalent refraction. Data are presented as mean ± standard deviation, unless otherwise indicated. Boldface values indicate statistical significance. * Probability values of linear mixed-effect models with age, sex, age at myopia onset, the number of parents with myopia, and initial SER adjustment for SER comparisons. † Probability values of linear mixed-effect models with age, sex, age at myopia onset, the number of parents with myopia, and initial axial length adjustment for axial elongation comparisons. | | | | | | | | |

| **Table S3**. Distributions of changes in spherical equivalent refraction and axial elongation in Phases 1 and 2. | | | | | | | | | | | | |
| --- | --- | --- | --- | --- | --- | --- | --- | --- | --- | --- | --- | --- |
|  | **SER changes** | | | | |  | **Axial elongation** | | | | | |
|  | **Phase 1** | |  | **Phase 2** | |  | **Phase 1** | |  | **Phase 2** | | |
| **Parameter** | **≤ 0.50 D** | **> 0.50 D** |  | **≤ 0.50 D** | **> 0.50 D** |  | **≤ 0.25 mm** | **> 0.25 mm** |  | **≤ 0.25 mm** | **> 0.25 mm** |  |
| SV/ESV, n(%) | 24 (35.8%) | 43 (64.2%) |  | 24 (35.8%) | 43 (64.2%) |  | 22 (32.8%) | 45 (67.2%) |  | 27 (40.3%) | 40 (59.7%) |  |
| P-PLARI, n(%) | 23 (65.7%) | 12 (34.3%) |  | 15 (42.9%) | 20 (57.1%) |  | 21 (60.0%) | 14 (40.0%) |  | 22 (62.9%) | 13 (37.1%) |  |
| P-NLARI, n(%) | 23 (63.9%) | 13 (36.1%) |  | 19 (52.8%) | 17 (47.2%) |  | 24 (66.7%) | 12 (33.3%) |  | 26 (72.2%) | 10 (27.8%) |  |
| N-PLARI, n(%) | 26 (68.4%) | 12 (31.6%) |  | 18 (47.4%) | 18 (52.6%) |  | 29 (76.3%) | 9 (23.7%) |  | 28 (73.7%) | 10 (26.3%) |  |
| N-NLARI, n(%) | 25 (75.8%) | 8 (24.2%) |  | 14 (42.4%) | 19 (57.6%) |  | 24 (72.7%) | 9 (27.3%) |  | 21 (63.6%) | 12 (36.4%) |  |
| SER = spherical equivalent refraction; SV = single-vision spectacle lens group; ESV = the extrapolated single-vision spectacle lenses group proposed by Brennan et al. [19] and Smotherman et al [18] in Phase 2; PLARI and NLARI = Lenslet-ARray-Integrated spectacle lens with lenslets of +3.00 D and −3.00 D addition powers, respectively; P-PLARI and N-NLARI = continued PLARI and NLARI, respectively in Phase 2; P-NLARI and N-PLARI = switched over to NLARI and PLARI, respectively in Phase 2. | | | | | | | | | | | | |

| **Table S4**. Average daily wearing hours of each group of participants. | | | | | | | | |
| --- | --- | --- | --- | --- | --- | --- | --- | --- |
| **Parameter** | **SV-PLARI  (n = 35)** | **SV-NLARI (n = 32)** | **P-PLARI (n = 35)** | **P-NLARI (n = 36)** | **N-PLARI (n = 38)** | **N-NLARI (n = 33)** | ***P* *** |  |
| Phase 1 | 13.8 ± 1.4 | 13.2 ± 1.5 | 13.2 ± 1.2 | 12.9 ± 1.6 | 13.0 ± 1.6 | 12.9 ± 2.0 | 0.21 |  |
| Phase 2 | 14.3 ± 0.7 | 14.1 ± 0.8 | 14.0 ± 0.7 | 14.3 ± 0.9 | 13.7 ± 1.0 | 14.1 ± 1.1 | **0.02** |  |
| *P* ^†^ | **0.02** | **0.004** | **< 0.001** | **< 0.001** | **0.01** | **< 0.001** |  |  |
| SV = single-vision spectacle lens group; PLARI and NLARI = Lenslet-ARray-Integrated spectacle lens with lenslets of +3.00 D and −3.00 D addition powers, respectively; SV-PLARI and SV-NLARI = switched over to PLARI and NLARI, respectively in Phase 2; P-PLARI and N-NLARI = continued PLARI and NLARI, respectively in Phase 2; P-NLARI and N-PLARI = switched over to NLARI and PLARI, respectively in Phase 2.  Data are presented as mean ± standard deviation. Boldface values indicate statistical significance.  * Probability values of one-way analysis of variance between groups.  ^†^ Probability values of paired t-test for differences between Phases 1 and 2 in each group. | | | | | | | | |

| **Table S5**. Changes in other parameters. | | | | | | | | |  |
| --- | --- | --- | --- | --- | --- | --- | --- | --- | --- |
| **Parameter** | **SV-PLARI**  **(n = 35)** | **SV-NLARI**  **(n = 32)** | **P-PLARI**  **(n = 35)** | **P-NLARI**  **(n = 36)** | **N-PLARI**  **(n = 38)** | **N-NLARI**  **(n = 33)** | ***P* *** | | |
| Changes in best-corrected visual acuity (logMAR) |  |  |  |  |  |  |  | | |
| Phase 1 | −0.03 ± 0.07 | −0.03 ± 0.06 | −0.02 ± 0.05 | −0.04 ± 0.05 | −0.02 ± 0.05 | −0.01 ± 0.06 | 0.60 | | |
| Phase 2 | −0.01 ± 0.06 | −0.02 ± 0.06 | −0.01 ± 0.06 | −0.01 ± 0.06 | −0.02 ± 0.06 | −0.02 ± 0.07 | 0.93 | | |
| *P* ^†^ | 0.37 | 0.34 | 0.28 | 0.12 | 0.87 | 0.87 |  | | |
| Changes in intraocular pressure (mmHg) |  |  |  |  |  |  |  | | |
| Phase 1 | 1.31 ± 1.66 | 1.86 ± 1.28 | 1.17 ± 1.71 | 1.17 ± 1.73 | 1.82 ± 1.97 | 1.16 ± 2.11 | 0.29 | | |
| Phase 2 | 0.10 ± 1.76 | 0.40 ± 2.09 | 0.21 ± 1.92 | 0.21 ± 1.64 | −0.37 ± 2.02 | 0.08 ± 1.65 | 0.62 | | |
| *P* ^†^ | **0.02** | **0.006** | 0.07 | 0.055 | **< 0.001** | **0.049** |  | | |
| Changes in steep keratometry (D) |  |  |  |  |  |  |  | | |
| Phase 1 | 0.01 ± 0.23 | 0.08 ± 0.29 | 0.06 ± 0.33 | −0.03 ± 0.20 | 0.05 ± 0.59 | 0.02 ± 0.25 | 0.83 | | |
| Phase 2 | 0.02 ± 0.28 | −0.04 ± 0.29 | −0.05 ± 0.25 | −0.04 ± 0.29 | −0.01 ± 0.59 | −0.05 ± 0.35 | 0.95 | | |
| *P* ^†^ | 0.77 | 0.19 | 0.22 | 0.85 | 0.76 | 0.47 |  | | |
| Changes in flat keratometry (D) |  |  |  |  |  |  |  | | |
| Phase 1 | −0.17 ± 0.15 | −0.16 ± 0.17 | −0.15 ± 0.19 | −0.15 ± 0.16 | −0.19 ± 0.15 | −0.14 ± 0.17 | 0.84 | | |
| Phase 2 | −0.11 ± 0.22 | −0.13 ± 0.17 | −0.15 ± 0.24 | −0.12 ± 0.22 | −0.08 ± 0.18 | −0.14 ± 0.27 | 0.83 | | |
| *P* ^†^ | 0.23 | 0.55 | 0.96 | 0.55 | **0.03** | 0.94 |  | | |
| Changes in corneal astigmatism (D) |  |  |  |  |  |  |  | | |
| Phase 1 | 0.18 ± 0.31 | 0.23 ± 0.38 | 0.20 ± 0.43 | 0.12 ± 0.26 | 0.32 ± 0.32 | 0.15 ± 0.33 | 0.17 | | |
| Phase 2 | 0.14 ± 0.36 | 0.08 ± 0.35 | 0.10 ± 0.34 | 0.08 ± 0.40 | −0.01 ± 0.35 | 0.09 ± 0.34 | 0.65 | | |
| *P* ^†^ | 0.63 | 0.21 | 0.35 | 0.69 | **0.001** | 0.52 |  | | |
| SV = single-vision spectacle lens group; PLARI and NLARI = Lenslet-ARray-Integrated spectacle lens with lenslets of +3.00 D and −3.00 D addition powers, respectively; SV-PLARI and SV-NLARI = switched over to PLARI and NLARI, respectively in Phase 2; P-PLARI and N-NLARI = continued PLARI and NLARI, respectively in Phase 2; P-NLARI and N-PLARI = switched over to NLARI and PLARI, respectively in Phase 2; logMAR = logarithm of the minimum angle of resolution.  Data are presented as mean ± standard deviation. Boldface values indicate statistical significance.  * Probability values of one-way analysis of variance between groups.  ^†^ Probability values of paired t-test for differences between Phases 1 and 2 in each group. | | | | | | | |  |  |

| **Table S6.** Characteristics and outcomes of studies of the 2-year efficacy of spectacle lenses for myopia control. | | | | | | | | | | | | | |
| --- | --- | --- | --- | --- | --- | --- | --- | --- | --- | --- | --- | --- | --- |
| **Author** | **Study design** | **Treatment** | **Baseline age (years)** | **Baseline SER (D)** | **N (Analyzed)** | | **SER Changes (D)** | | **Axial elongation (mm)** | | **Efficacy** | |  |
|  |  |  |  |  | **Treated** | **SV** | **Treated** | **SV** | **Treated** | **SV** | **SER changes (D)** | **Axial elongation (mm)** |  |
| Lam et al. [8] | 2-year RCT | DIMS | 8 to 13 | −5.00 to −1.00 | 79 | 81 | −0.38 | −0.93 | 0.21 | 0.53 | **0.55** | **0.32** |  |
| Bao et al. [7] | 2-year RCT | HAL | 8 to 13 | −4.75 to −0.75 | 54 | 50 | −0.66 | −1.46 | 0.34 | 0.69 | **0.80** | **0.35** |  |
|  |  | SAL | 8 to 13 | −4.75 to −0.75 | 53 | 50 | ­1.04 | −1.46 | 0.51 | 0.69 | **0.42** | **0.18** |  |
| Liu et al. [22] | 2-year Retrospective | DIMS | 6 to 16 | < 0 | 735 | 735 | −0.88 | −1.23 | - | - | **0.35** | - |  |
| SER = spherical equivalent refraction; DIMS = defocus incorporated multiple segments; HAL = highly aspherical lenslets; SAL = slightly aspherical lenslets.  Boldface values indicate statistical significance. | | | | | | | | | | | | | |
